# Evaluation of the Patient-Reported Outcome Measures (PROMs) With Temporary Skeletal Anchorage Devices in Fixed Orthodontic Treatment: A Systematic Review

**DOI:** 10.7759/cureus.36165

**Published:** 2023-03-15

**Authors:** Mudar Mohammad Mousa, Mohammad Y Hajeer, Kinda Sultan, Wael H. Almahdi, Jacqueline Bashar Alhaffar

**Affiliations:** 1 Department of Orthodontics, University of Damascus Faculty of Dentistry, Damascus, SYR; 2 Department of Periodontics, University of Damascus Faculty of Dentistry, Damascus, SYR; 3 Department of Oral and Maxillofacial Surgery, University of Damascus Faculty of Dentistry, Damascus, SYR

**Keywords:** questionnaire, functional impairment, speech, pain, patient-reported outcome measures, headgear, nance buttons, transpalatal arches, mini-implants, anchorage

## Abstract

Analysis of patient-reported outcome measures (PROMs) is essential to ensure that the skeletal and traditional anchoring methods are appropriately and effectively utilized in the context of patient acceptance and satisfaction. This review's objective was to assess the available data on the levels of discomfort, soft-tissue irritation, functional impairment, and other patient-reported outcomes related to the usage of mini-implants in the context of fixed orthodontic treatment for adult patients. A total of seven electronic bibliographic databases were searched between January 1995 and February 2022. Moreover, a manual search was done in the selected orthodontic journals. This systematic review (SR) covered cohort studies, retrospective studies, randomized clinical trials (RCTs), and controlled clinical trials (CCTs) that studied the use of mini-implants, mini-plates, or onplants as anchorage devices on patients receiving orthodontic treatment. The risk of bias was assessed using Cochrane’s risk of bias tool (RoB2 tool). Three RCTs and two cohorts were included in this SR with a total of 468 patients. Three of the four included studies were at high risk of bias. The pain level was in the “mild-to-moderate” category on the first day following the insertion of mini-implants, then decreased to a mild level from the fifth day to the seventh day of insertion (mean values are 36.61, 16.36, and 11.33, respectively). The levels of functional impairments were found to be located between the “mild-to-moderate” and “moderate” categories after the placement of mini-plates and intermaxillary fixation screws, while they experienced a mild level with mini-implants. The greatest pain levels were found after the insertion of the temporary anchorage devices (TADs) and then decreased until they became mild or disappeared completely after one month. Speaking, chewing, and cleaning difficulties were more problematic when using TADs compared to conventional anchorage. To obtain good evidence in this area, more high-quality RCTs are needed.

## Introduction and background

Planning and preparing anchorage before starting tooth movement is essential to avoid unwanted movement and complications [1]. Traditionally, orthodontists have used a variety of methods (intraoral/extraoral appliances) to control anchorage and achieve desired tooth movement [2], such as headgears, lingual holding arches, transpalatal arches (TPAs) with or without Nance buttons, intermaxillary elastics, and the inclusion of second molars in the anchoring units [3].

Each method has its advantages and disadvantages. For example, the success of headgear depends on the patient's cooperation, and facial injuries may occur [4]. In addition, although TPA is widely used, it is ineffective in cases with severe anchoring demands [5], and TPA with the Nance button may be associated with inflammation of the soft tissues under the acrylic button [6]. For this reason, temporary anchorage devices (TADs) have been introduced and have become widely used as intraoral devices that can provide maximum to absolute anchorage and reduce the need for patient cooperation during orthodontic treatment [7].

Mini-implants are used in orthodontic treatment in various ways, including direct and indirect anchorage [8], teeth intrusion, molar uprighting, and distalization [9]. However, the most common usage is en-masse retraction of anterior teeth [10,11]. Among the many advantages are small size, ease of insert and handling, high success rate, good tolerance, immediate loading, relatively low cost, and more comfortable procedure for the patient [12,13].

However, the success of orthodontic treatment depends on whether it is easy, simple, and free of complications related to pain, discomfort, or functional impairment [14,15]. Many procedures during orthodontic treatment can cause pain and discomfort, from separation [16] through archwire placement [17], ending with bracket and adhesive removal [18]. The orthodontics treatment may fail if focused only on improving the occlusal and functional aspects without considering the patient's satisfaction. Therefore, in addition to the efficacy of the treatment offered, there is a need to evaluate patient tolerance to the treatment steps and the related quality of life during the orthodontic treatment course [19].

Mirhashemi et al. found that the level of pain at one hour following mini-implant placement was moderate [20], whereas Sobouti et al. reported that the pain level was mild to moderate at the same assessment time (i.e., one hour following insertion) [21]. However, it was perceived as mild in the study by Kuroda et al. [22].

A systematic review (SR) was published by Giudice et al. [23], which evaluated the complications caused by the use of mini-implants as a primary outcome, but this review has several drawbacks as 50% of the articles included were case reports and case series, which focused especially on general complications arising from mini-implants such as root perforation and loss of tooth vitality, periarticular lesion, and maxillary sinus perforation, among others. In addition, patient-reported outcomes were not systematically studied, while the authors considered the pain associated with using mini-implants only as a side effect and a secondary outcome.

Therefore, the current review aimed to critically evaluate the existing evidence on the pain, discomfort, functional impairment, or soft-tissue irritation associated with the moderate- or long-term use of mini-implants in fixed orthodontic treatment for the different types of tooth movement presented. The focused review questions were: "What are the pain, discomfort, functional impairment, soft-tissue irritation, and other patient-reported outcomes associated with long-term use of mini-implants in the context of fixed orthodontic treatment in adult patients?"

## Review

Scoping search

Before starting this SR, a scoping PubMed search was conducted to ensure that there were no previous SRs and also to check for potentially eligible trials. The result showed no SRs on the same topic, and two potentially eligible studies were presented.

Eligibility criteria

The Participants/Intervention/Comparison/Outcome/Study Design (PICOS) Framework

Participants: Adult patients aged 16-30 years of either sex and any ethnic group who were undergoing orthodontic treatment with insertion of a mini-implant and/or mini-plate and/or onplant at any site.

Intervention: Fixed orthodontic treatment using mini-implants, mini-plates, and/or onplant as anchorage devices.

Comparison: Fixed orthodontic treatment with a conventional anchorage system (transpalatal arch with or without Nance button, headgear, lingual arch, etc.) or another type of mini-implants/mini-plates, which was not employed in the main interventional group.

Outcome measures: Evaluation of patients' perception of pain and discomfort, functional impairment, or swelling as measured by a verified scale. The authors developed or validated questionnaires (open-/closed-ended questions) to assess complications and oral health-related quality of life.

Study design: Randomized clinical trials (RCTs) and controlled clinical trials (CCTs), cohort studies, and retrospective studies published between January 1995 and February 2023 with no limitation on the language were used.

Exclusion criteria: Animal studies, in vitro studies, case reports, case series reports, finite element analysis studies, editorials or personal opinions, reviews, and technique description articles were excluded. In addition, this review did not include studies with no control groups or a control group of non-treated subjects. Any study with a statistical analysis including less than 10 patients was also excluded.

Information sources

The keywords used in the search strategy are given in Appendix 1. The primary search was conducted using PubMed®, Google™ Scholar, Scopus®, Embase®, OpenGrey, Cochrane Library, and Web of Science by two review authors (MMM and MYH). Databases between January 1995 and January 2023 were searched. A hand search of the bibliography of all included articles was done to identify additional relevant articles.

Search strategy and study selection

The selection of articles was made in two steps. In step one, the two reviewers (MMM and MYH) independently reviewed the titles and abstracts of the articles identified in the electronic search. In step two, the full texts of all articles that could be included in the review were evaluated by the same two reviewers. Articles that did not meet the inclusion criteria were excluded from the review. Conflicts were resolved by the reviewers through discussion until an agreement was reached.

Data collection process

Two reviewers (MMM and MYH) extracted data from the included studies and arranged them into tables that included general information (authors' names, year of publication, and study setting), methods (study design and questionnaire type), participants (sample size, age, and type of malocclusion), mini-implant characteristics (length, diameter, location, and position), and outcomes (administration time, measurement method, results of assessments, and differences between groups).

Assessment of risk of bias in individual studies

Using the Cochrane Risk of Bias Tool, two reviewers (MMM and MYH) evaluated the quality of the included studies. The judgments of the two reviewers were then compared; if there were disagreements, the reviewers resolved them through discussion with the third author (KS) until a consensus was reached. The Risk of Bias 2.0 (RoB-2) tool was used to assess the risk of bias for RCTs, and the Risk of Bias in Nonrandomized Studies - of Interventions (ROBINS-I) tool was used for nonrandomized controlled trials [24]. The following domains were rated as low, high, or having some concerns about bias in randomized trials: the randomization process, deviations from intended interventions, missing outcome data, outcome measurement, and selection of reported outcomes. The following factors were used to assess the overall risk of bias: low risk of bias - if all domains were rated as low risk of bias; some bias concerns - if at least one or more domains were identified as having some bias concerns, and high risk of bias - if at least one or more domains were identified as having a high risk of bias.

Results

The Flow of Study Selection and Inclusion in this Systematic Review

**Figure 1:**
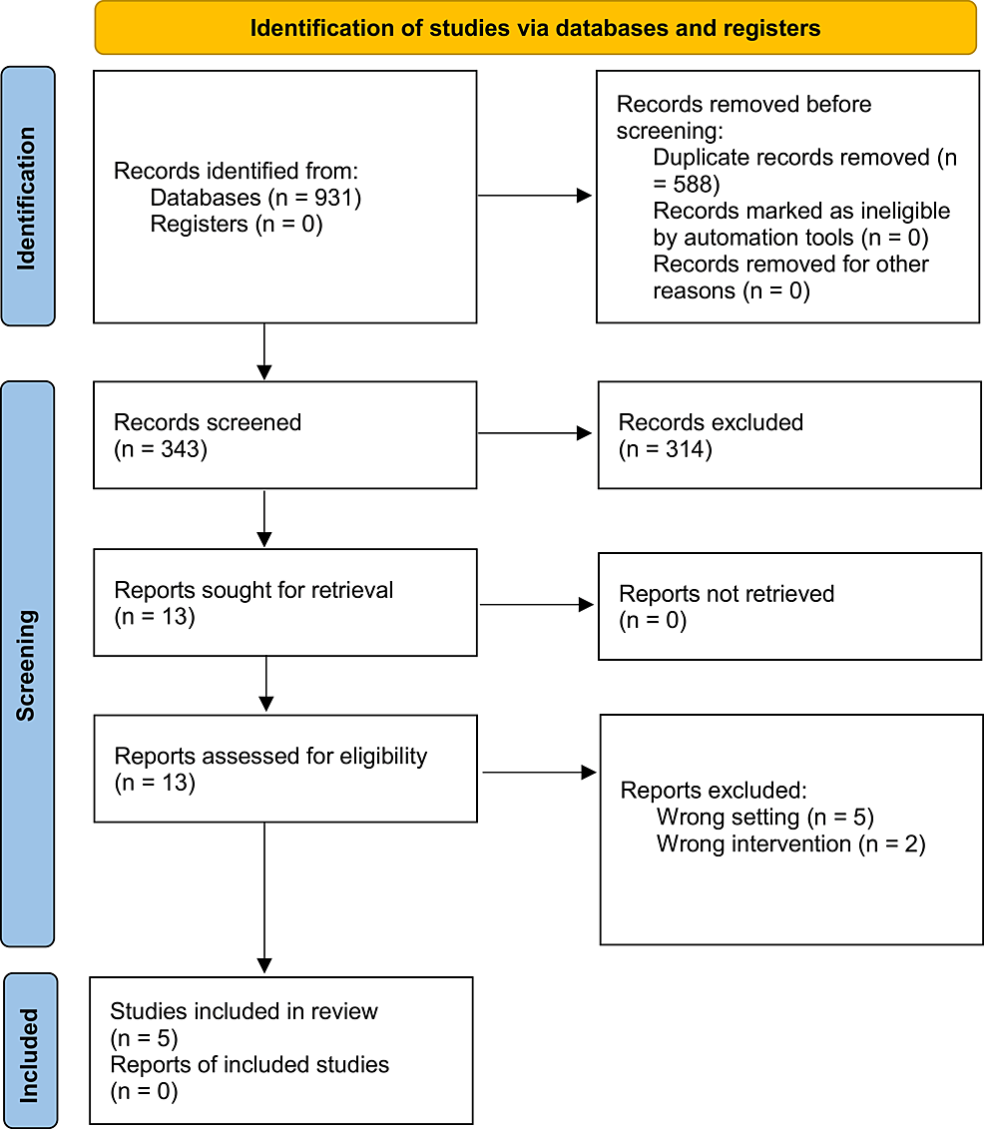
Preferred Reporting Items for Systematic Reviews and Meta-Analyses (PRISMA) flow diagram of the studies' identification, screening, and inclusion into this review

After the electronic screening, 931 articles were identified, and 343 articles were reviewed after removing duplicate references. Titles and abstracts were carefully screened for suitability. Subsequently, all articles that did not meet the eligibility criteria were excluded. The full text of 14 articles was reviewed in detail. As a result, five articles were included in the SR [2,22,25-27]; the Preferred Reporting Items for Systematic Reviews and Meta-Analyses (PRISMA) flow diagram of the studies' identification, screening, and inclusion into this review is presented in Figure 1.

Characteristics of the Included Studies

The characteristics of the five included studies of the SR are summarized in Table 1. There were three completed RCTs [2,26,27] and two cohorts [22,25], with 468 patients, which were included in this SR. Age ranged from 12.28 to 34.4 years. All studies had male and female subjects in their samples (135 males and 213 females). In one study, the number of females was less than that of males [27]. In three studies, the number of males was less than that of females [2,22,25]. In one study, the number of males was equal to that of females [26].

**Table 1:** Characteristics of included studies in the systematic review RCT: Randomized clinical trial; PGD: Parallel-group design; Retro: Retrospective; G: Group; TPA: Transpalatal arch; M: Male; F: Female; VAS: Visual analog scale; NRS: Numeric rating scale.

Study/setting	Study design	Treatment comparison/movement type	Number of patients	Mini-implants properties	Assessment method	Follow-up	Outcomes
Mousa et al., 2022 [2]	RCT PGD	G A: Mini-implant; G B: TPA Class II division I	N = 38; G A: 19; G B: 19	Length: 8 mm; diameter, 1.6 mm	4-point Likert scale	4 weeks	Pain, swelling, difficulty in chewing, speech difficulty, and difficulty in cleaning
Sandler et al., 2014 [27]	RCT PGD	G A: Mini-implant; G B: Nance button; G C: Headgear. Patients needed maximum anchorage.	N = 78; G A: 27; G B: 26; G C: 25	Length: 8 mm; diameter, 1.6 mm	6-point Likert scale	2 weeks	G A/B: Levels of comfort and discomfort. G C: Comfort and convenience.
Feldmann et al., 2012 [26]	RCT	G A: Onplant or orthosystem implant; G B: Headgear; G C: TPA	N = 120; G A: 60; G B: 30; G C: 30	No information given	VAS binary response (yes/no) open-ended question; 4-point scale	6 weeks in the retention phase	Discomfort, analgesic consumption, and jaw function impairment
Lee et al., 2008 [25]	Cohort	Extraction separation initial alignment mini-implants	N = 37	Length: 7 mm; diameter: 1.3–1.4 mm	VAS	4 weeks following the placement of the mini-implants	Patients’ expectations of pain and pain experienced intraoperatively
Kuroda et al., 2007 [22]	Retro cohort	G A: Intermaxillary fixation screw; G B: Mini-implant; G C: Mini-plate	N = 75	G A: diameter: 2.0 or 2.3 mm; length: 7 or 11 mm. G B: diameter: 1.3 mm; length: 6, 7, 8, 10, and 12 mm	VAS	2 weeks after insertion	Pain, swelling, difficulty in chewing, speech difficulty, and difficulty in toothbrushing

Four studies investigated self-drilling mini-implants [2,22,25,27], and the mini-implants differed in their dimensions (diameter and length). The diameter ranged from 1.3 mm [22,25] to 1.6 mm [2,27], and the length was either 7 mm [25] or 8 mm [2,27]. However, one study used mini-implants of different lengths, ranging from 6 to 12 mm [22]. One trial used an intermaxillary fixation screw with two lengths, 7 or 11 mm [22]. Mini-implants were placed in the interdental areas mesial and distal to all first molars in one trial [25]; it was placed in the area of premolars and/or molars [22]. In another study, the mini-implants were placed between the upper first molar and second premolar [2]. One study examined mini-plates inserted with two to three screws where the anchor appearance was desired [22] but did not specify exactly where these mini-plates were inserted. Onplants were inserted at the mid-palatal area in one trial [26]. In the comparative studies where the comparison group involved non-TAD-based methods, two trials used the headgear [26,27], two included trials compared with the use of a TPA [2,26], and one trial included the use of a Nance button in conjunction with a palatal arch [27].

Visual analog scales (VAS) were used as the scoring method in three studies [22,25,26]; the Likert scale with 6 points was used in one trial [27] and with 4 points was used in two trials [2,26]; open-ended and dichotomous questions were used in one trial [26]. Follow-up time ranged from two weeks [22,27], one month [2], six weeks [26], to 28 months after anchorage device insertion [25]. Pain levels were assessed in all studies [2,22,25,26] except one study [27]. Four studies also investigated discomfort [2,22,26,27], except for one trial [25]. Swelling, difficulty chewing, difficulty brushing teeth, and speech difficulties were investigated in two studies [2,22]. Finally, analgesic consumption was investigated in one study [26].

Risk of Bias of Included Studies

**Figure 2:**
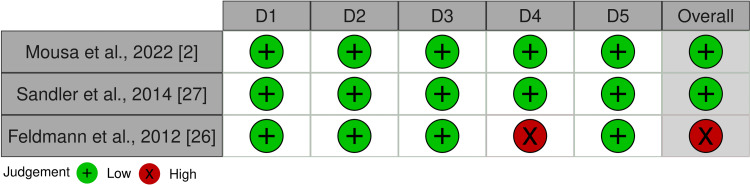
Risk of bias summary of RCTs: the review authors’ judgments about each item of the risk of bias for the included studies D1: Randomization process; D2: Deviations from intended interventions; D3: Missing outcome data; D4: Measurement of the outcome; D5: Selection of the reported result; RCTs: Randomized clinical trials.

**Figure 3:**
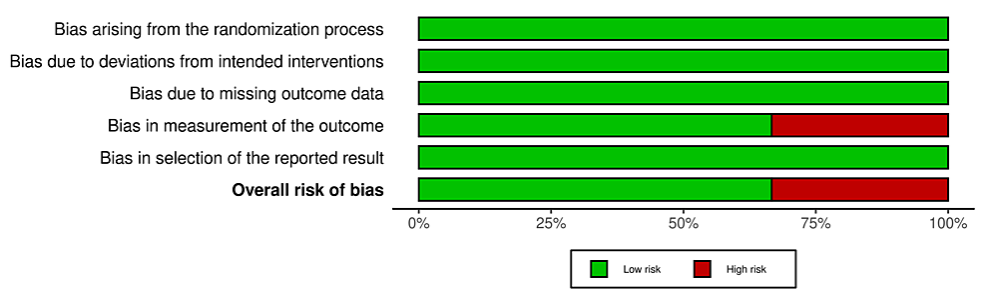
The overall risk of bias score for each field of RCTs: the review authors’ judgments about each item of the risk of bias, presented as percentages across all the studies included RCTs: Randomized clinical trials.

**Figure 4:**
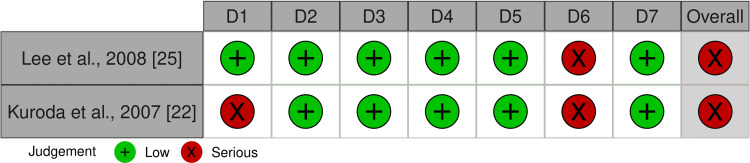
Risk of bias summary of nonrandomized studies: the review authors’ judgments about each item of the risk of bias for the included studies D1: Bias due to confounding; D2: Bias in the selection of participants for the study; D3: Bias in the classification of interventions; D4: Bias due to deviations from intended interventions; D5: Bias due to missing data; D6: Bias in the measurement of outcomes; D7: Bias in the selection of the reported result; RCTs: Randomized clinical trials.

**Figure 5:**
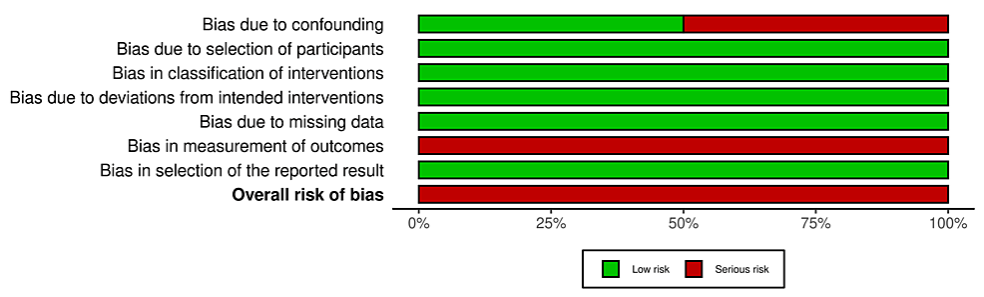
The overall risk of bias score for each field of nonrandomized studies: the review authors’ judgments about each item of the risk of bias, presented as percentages across all the studies included

The overall risk of bias in the included RCTs is summarized in Figures 2, 3. Two RCTs were classified as having a “low risk of bias” [2,27]. The other RCT was classified as having a “high risk of bias” [26]. Bias in measuring the outcome was the reason for the high risk of bias. The risk of bias assessment of the cohort studies is shown in Figures 4, 5. The two studies had a “serious risk of bias” [22,25]. Reasons beyond a serious risk of bias include bias in measuring the outcome. Additional details about assessing the risk of bias are provided in Appendix 2.

Effects of intervention

Pain

Mousa et al., in their RCT, evaluated pain levels associated with the use of mini-implants in comparison with TPA [2], and they found that after 24 hours, the pain perception in the mini-implant group was moderate to severe, then reduced to mild or moderate after seven days, two weeks, and one month (mean values were 3.26, 2.42, 2.68, 1.79 and 1.58, respectively). However, after 24 hours, in the TPA group, pain perception was mild to moderate, then decreased to mild after three days, seven days, and two weeks (mean values were 1.42, 1.26, 1.26, 1.21, and 0.77, respectively). In the mini-implant group, the pain sensation was significantly higher (P = 0.001) [2]. In the retrospective collection of patients' responses in the study by Kuroda et al. [22], the greatest level of pain was at one hour after insertion of the mini-plates and intermaxillary fixation screw (mean VAS values: 66.4 and 65.7, respectively), after which the pain level became mild from the seventh to the 14th day after insertion.

On the other hand, the pain intensity was mild with the use of mini-implants at the first hour after insertion, then disappeared on the seventh day (mean VAS values: 19.5 and 0, respectively). The difference in pain level between mini-implants and mini-plates or intermaxillary fixation screws was statistically significant immediately at seven days after surgery (P < 0.05), after which the difference became insignificant [22]. Feldmann et al., in their three-arm trial, evaluated the PROMs associated with the use of onplants, headgear, or transpalatal bar and found that patients experienced moderate pain levels two days after the onplant placement in the incisors' and molars' areas (median VAS values: 46, and 14, respectively) [26]. However, the pain level decreased from the third day and reached a mild level on the seventh day in the incisor and molar areas (median VAS values: 2.3 and 1.5, respectively). After six weeks, the pain level decreased to zero and remained at this level until the end of the treatment. However, there were no significant differences in the incisors’ pain intensity between the onplants, the headgear, and the TPA groups. On the contrary, the molars' pain intensity was significantly greater at two, three, four days, and six weeks after insertion in the TPA group compared to the other two groups [26]. Lee et al. evaluated the pain levels associated with using mini-implants at different stages of the orthodontic treatment, including molar separation, premolar extraction, and initial tooth alignment [25]. When the mini-implants were inserted after the initial alignment stage, the pain was mild to moderate on the first day following the insertion (mean VAS value: 36.61), then decreased to reach a mild level on the fifth and seventh day of insertion (mean VAS values: 16.36, and 11.33, respectively). Overall pain was significantly greater with initial tooth alignment than with the other orthodontic procedures, i.e., mini-implant insertion, molar separation, and premolar extraction (P < 0.05) [25].

Discomfort

Sandler et al. found that the levels of comfort assessed on a six-point Likert scale were similar between the TADs’ group and the combined Nance button-transpalatal arch group at placement time (mean values: 4.41 and 4.62, respectively) and during the first three days (mean values: 3.73, and 3.46, respectively) [27]. A similar level of comfort was also noted with the removal of the TADs as with the removal of the Nance button (mean values: 4.25 and 4.31, respectively) and in the first three days after removal (mean 6 values: 4.81 and 4.92, respectively) [27]. When comparing skeletal anchorage with transpalatal arch or headgear in the study by Feldmann et al., patients experienced significantly less discomfort or soreness in the headgear group on the third day (P = 0.030), after five days (P = 0.010), after six weeks (P = 0.028), after leveling/aligning (P = 0.036), and after space closure [26]. On the contrary, there were no significant differences between groups in the discomfort of jaw and tooth tension [26].

Swelling

Mousa et al. found that swelling was at a mild level in 73.68% of patients at the site of the mini-implant 24 hours after it was inserted. Then, at two weeks and one month, there was a significant decrease (P = 0.002, P = 0.004, respectively). During each assessment period, none of the patients had severe or moderate swelling around the TPA. After two weeks, all patients' perceived swelling in adjacent tissues was near zero. In the study by Kuroda et al., after the placement of mini-plates and intermaxillary fixation screws, the level of discomfort of swelling was moderate to severe compared to a mild level with mini-implants [22].

Functional Impairments

Mousa et al. studied speech discomfort, chewing difficulty, and cleaning difficulty using mini-implants compared to TPA [2]. They found that 68.42% of the patients in the mini-implant group reported moderate to severe discomfort while eating 24 hours after the mini-implant implantation; this percentage significantly dropped after three days (P = 0.006). However, all patients reported no or mild discomfort after one month of mini-implant and TPA placement. [2]. In addition, 47.37% of patients experienced moderate speech discomfort after 24 hours following the insertion of the mini-implant and TPA. However, the discomfort decreased to "very mild" or "mild" after three days, one week, and one month [2]. Moreover, after 24 hours, 68.43% of patients reported moderate to severe cleaning difficulties around the mini-implants, which reduced after two weeks and reached 21.05% after one month. After 24 hours, about 36.84% of patients in the TPA group experienced mild cleaning difficulties. After one month, 94.74% of patients had no cleaning difficulties [2]. Using self-reported questions concerning functional jaw impairment during orthodontic treatment, Feldmann et al. found that jaw function and daily life limitations were low to moderate with the three anchorage techniques (i.e., onplants, headgear, and transpalatal) at all assessment times until the end of treatment [26]. In addition, Kuroda et al. found that patients experienced a moderate level of difficulty in chewing and a mild-to-moderate level of speech difficulty after placement of mini-plates and intermaxillary fixation screws, while they experienced a mild level of difficulty in chewing and speaking with mini-implants [22].

Discussion

Many previous SRs have studied skeletal anchoring devices' success rates, benefits, and clinical efficacy [28,29]. However, no SR specifically has addressed the pain and discomfort associated with these methods. Hence, this review is important as this is the first SR that tries to critically appraise the existing evidence on PROMs related to the use of mini-implants in the context of fixed orthodontic therapy for the various types of tooth movement reported.

Pain Levels

There is an agreement between the studies that greater pain levels were noted with the use of mini-plates and onplants, where the pain levels were found between the “moderate” and “moderate-to-severe” categories after insertion [22,30] and then reached mild values after seven days. These high levels can be explained by the invasive nature of installing these types of TADs, which involves the need to elevate and suture flaps, leading to tissue trauma and edema and, thus, higher pain levels.

On the other hand, pain levels associated with mini-implants ranged between mild and “mild to moderate,” especially in the first three days, then it decreased and reached zero after seven days [22,25]; this may be attributed to the minimal surgical invasion in which mini-screws were inserted without flap surgery, so it may be more comfortable for the patients.

Only one study compared pain levels between TADs and conventional anchorage systems. Feldmann et al. found that the TPA group experienced significantly higher pain intensity in the molar region than the onplant and headgear groups two, three, four days, and six weeks after insertion [26]. This can be explained by the greater ability of the onplants to anchor the molars, whereas TPA allows for a greater range of movement and, thus, more continuous and greater pain. Furthermore, because the headgear is only worn for a portion of the day, the force application is intermittent, and the pain is lower.

Discomfort Levels

The level of discomfort was found to be located in the "mild-to-moderate" category with the use of mini-implants and combined Nance button-TPA at the time of insertion and removal as well as three days later in the study by Sandler et al. [27]. The reason for this seems to be the contribution of the combined Nance button-TPA in reducing the space for the tongue and the direct contact between them during the processes of chewing, swallowing, and speaking. On the other hand, discomfort associated with using mini-implants is due to the pain and swelling in the surrounding tissues and uncomfortable contact between the cheeks and the two protruding implants.

Swelling

The level of discomfort with the use of mini-plates and intermaxillary fixation screws was in the “moderate-to-severe” category compared to the mild level with the use of mini-implants [22]. Mini-plates and intermaxillary fixation screws inserted through movable soft tissue might cause edema around the implantation site following flap surgery or infection during treatment. In contrast, mini-implants inserted at the attached gingiva without incision are less prone to cause infection and irritation, so they have less discomfort.

In contrast, the level of swelling with the use of mini-implants reached the moderate category in the study by Mousa et al. [2]. The discrepancy between these two studies can be explained by the differences in retraction methods and the use of different diameters of mini-implants. Mousa et al. used mini-implants of 1.8 mm diameter with en-masse retraction [2], whereas Kuroda et al. used mini-implants of 1.3 mm diameter with canine retraction [22]. This could shorten the coil spring or power chain extension from the mini-implant to the canine bracket hook in the two-step retraction. Therefore, in the en-masse retraction, the power chains continuously exerted pressure on the gingiva, particularly in the maxillary corners, which occasionally resulted in embedding in the soft tissue around them.

Functional Impairments

Two trials examined the functional impairments with the use of mini-implants, where the patients experienced a mild level of speech and chewing difficulties after 14 days [22] and one month of use [2], while it was at mild-to-moderate and moderate categories with the mini-plates and intermaxillary fixation screws [22]. This can be explained by the use of smaller diameter mini-implants without incisions or surgical flap elevation, which has reduced the sensation of pain and swelling that causes discomfort during chewing and speech.

The level of cleaning difficulty was lower around the TPA than around the mini-implants [2]. This can be explained by the swelling and pain around the mini-implant and the accumulation of food debris due to many factors, such as braided wire, power chains, and hooks welded to the archwire.

Limitations of the current systematic review

The current SR found only three RCTs and two cohorts. Because of the variety of anchoring systems used, the types of malocclusions treated, the assessment methods, and the assessment times, a meta-analysis was not conducted in this review, and no precise pooled estimate could be derived. The PROMs related to swelling, difficulty chewing, difficulty brushing teeth, and speech difficulties were addressed in only two of the included studies. Moreover, jaw functional impairments and analgesic consumption associated with the use of TADs were reported in only one study. Furthermore, no study evaluated the levels of cheek irritation or the consequences of gingival soft-tissue impingement during force application by power chains or coil springs. These untoward effects are not objectively evaluated in the included studies.

## Conclusions

The highest pain intensity was noted after the insertion of TAD and ranged from the “moderate” and “moderate-to-severe” categories (whether mini-plates, mini-implants, intermaxillary screws, or onplants were used). These levels decreased after one week, reached “mild” levels, or disappeared completely after one month. In addition, the levels of discomfort of swelling reached the “moderate-to-severe” category during the first week of using TAD and then decreased or disappeared within one month. In addition, functional impairment related to skeletal anchorage devices reached the "moderate-to-severe" category; these levels decreased to a mild level after 14 days and one month of use. On the other hand, using conventional anchorage systems was most likely less problematic than TADs.

In this SR, most included studies were at “high risk” of bias. Therefore, more well-conducted RCTs are needed to compare the PROMs accompanied by TADs and the traditional anchoring systems. Additionally, future research should consider the follow-up period and standardization of the evaluation times and the TADs used (type, size, and position).

## Appendices

Appendix 1 

**Table 2:** Keywords used in the search strategy

Components of the search strategy	Keywords or medical terms
Orthodontics	Orthodontic treatment, orthodontic therapy, orthodontics, tooth movement, orthodontic tooth movement, tooth displacement.
Patient-reported outcome measures	PROMs, pain, discomfort, swelling, bleeding, restriction, mastication, chewing, eating, articulation, speech, limitation, tension, pressure, patient-reported outcome measures, problem, visual analog scale, VAS, numerical rating scale, NRS, Likert scale.
Anchorage	Anchorage, skeletal anchorage, maximum anchorage, absolute anchorage, traditional anchorage, temporary anchorage devices, TADs.
Devices used for anchorage	Transpalatal arch, TPA, transpalatal bar, Nance button, headgear, mini‑plate, mini‑screw, micro-screw, mini‑implant, micro‑implant, ortho-implant, onplant.

Appendix 2 

**Table 3:** Risk of bias of the included trials ROB-2: Risk of Bias; ROBINS-I: Risk of Bias in Non-randomized Studies - of Interventions.

Risk of bias assessment for randomized controlled trials according to the ROB-2 tool												
Study	Randomization process		Deviations from intended interventions		Missing outcome data			Measurement of the outcome		Selection of the reported result		Over-all bias
Mousa et al., 2022 [2]	Low risk: “Minitab® version was used to generate the random number sequence...”		Low risk: We judge that the outcome is not likely to be influenced by a lack of blinding.		Low risk: No dropouts were reported.			Low risk: “Blinding was limited to the data analysis only…”		Low risk: The protocol ID (NCT05652244) and the outcomes mentioned in the protocol have been reported.		Low risk
Sandler et al., 2014 [27]	Low risk: “The randomization was based on a computer-generated pseudorandom…”		Low risk: “The clinicians and the patients were blinded to the allocation sequence…”		Low risk: Seven patients had withdrawn, which is equivalent to 7% of the sample size. The author assumed that a dropout rate of 20% was added to the final sample size.			Low risk: “Assessment was blind because it was impossible to distinguish between the groups…”		Low risk: The protocol ID (NCT00995436) and the outcomes mentioned in the protocol have been reported.		Low risk
Feldmann., 2012 [26]	Low risk: “The allocation sequence was computer generated…”		Low risk: We judge that the outcome is not likely to be influenced by a lack of blinding.		Low risk: Six patients were excluded, which is equivalent to 5%.			High risk: No information about outcome assessors blinding.		Low risk: The protocol was not registered. But the pre-defined outcomes mentioned in the methods section seemed to have been reported.		High risk
Risk of bias for non-randomized controlled trials according to the ROBINS-I tool												
Study	Bias due to confounding	Bias in the selection of participants for the study		Bias in the classification of interventions		Bias due to deviations from intended interventions	Bias due to missing data		Bias in the measurement of outcomes		Bias in the selection of the reported result	Over-all bias
Lee et al., 2008 [25]	Low risk: No confounding expected	Low risk: All participants who would have been eligible for the target trial were included in the study. For each participant, the start of follow-up and the start of intervention coincided.		Low risk: Intervention status is well defined, and intervention definition is based solely on information collected at the time of intervention.		Low risk: There were no deviations from the intended intervention beyond what would be expected in usual practice.	Low risk: Outcome data were available for all participants. No participants were excluded due to missing data.		Serious risk of bias: No information about outcome assessors blinding, and the results may be influenced by knowledge of the intervention received by study participants.		Low risk: The measurements and analysis of selected variables were reported in the study.	Serious risk of bias
Kuroda et al., 2007 [22]	Serious risk of bias: Pain and discomfort were measured in the retrospective method.	Low risk: All participants who would have been eligible for the target trial were included in the study.		Low risk: Intervention status is well defined, and intervention definition is based solely on information collected at the time of intervention.		Low risk: There were no deviations from the intended intervention beyond what would be expected in usual practice.	Low risk: Outcome data were available for all participants. No participants were excluded due to missing data.		Serious risk of bias: No information about outcome assessors blinding, and the results may be influenced by knowledge of the intervention received by study participants.		Low risk: The measurements and analysis of selected variables were reported in the study.	Serious risk of bias
